# Urinary Silicon Excretion in Relation to Lactation and Bone Mineral Density — a Longitudinal Study Post-partum

**DOI:** 10.1007/s12011-024-04175-8

**Published:** 2024-04-24

**Authors:** Catarina Magnusson, Hanna Augustin, Ravin Jugdaohsingh, Jonathan J. Powell, Lena Hulthén, Maria Ransjö

**Affiliations:** 1 Department of Orthodontics, Institute of Odontology, The Sahlgrenska Academy, University of Gothenburg, PO Box 450, 405 30 Gothenburg, Sweden; 2 Department of Internal Medicine and Clinical Nutrition, The Sahlgrenska Academy, University of Gothenburg, Gothenburg, Sweden; 3 Biomineral Research Group, Department of Veterinary Medicine, University of Cambridge, Cambridge, UK; 4 Department of Odontology, Umeå University, Umeå, Sweden

**Keywords:** Silicon, Bone minerals, Lactation, Pregnancy, Bone metabolism

## Abstract

Silicon (Si) may be a mineral beneficial for bone health. Pregnancy and lactation have major impacts on maternal bone metabolism as bone minerals, including calcium (Ca), are required for growth of the foetus and for milk production. Like urinary Ca excretion, Si excretion has been reported to be high in pregnant women, but there are no data post-partum and during lactation. The aim of the present study was to investigate the urinary excretion of Si (U-Si), from the third trimester of pregnancy until 18 months post-partum, and in relation to the length of lactation, to determine if changes in U-Si are associated with changes in areal bone mineral density (aBMD). This longitudinal study included 81 pregnant women, of whom 56 completed the study. Spot urine samples were collected at the third trimester and at 0.5, 4, 12, and 18 months post-partum and were analysed for Si and Ca by ICP-OES. The aBMD was measured post-partum at lumbar spine and femoral neck by dual-energy x-ray absorptiometry. Women lactating for 4–8.9 and ≥ 9 months had significantly higher U-Si at 4 months post-partum, compared with the third trimester. No significant longitudinal differences in U-Si were found after correcting for creatinine. Changes in U-Si and in aBMD were not correlated, except at the lumbar spine from 0.5 to 12 months post-partum in the women lactating for 4–8.9 months. Taken together, our results suggest that there is a possibility that U-Si increases post-partum in women lactating for 4 months or longer, although it is not related to changes in aBMD.

**Supplementary Information:**

The online version contains supplementary material available at 10.1007/s12011-024-04175-8.

## Introduction

Research over the last four decades have implied that silicon (Si) may contribute to the development and maintenance of a healthy bone and connective tissues [1]. Positive association between dietary Si intake and bone mineral density (BMD) in men and pre-menopausal women was found in a large-scale cross-sectional study [2]. Drinking water and plant/cereal-based foods are the main sources of Si and, although most of the absorbed Si is readily eliminated, some is retained [3, 4]. Concentrations of Si in human tissues are not established, but have been measured in the vertebral column, confirming trace amounts of Si in human bone [5]. However, animal studies have revealed higher abundance of Si in bone and connective tissues compared to other tissues [6, 7].

Studies from the 1970s on growing rats and chicks receiving a Si depleted diet were the first to suggest an impact of Si on bone since the animals stunted in growth and had severe bone deformities [8, 9]. However, it is very unlikely that humans would develop chronic Si deficiency with bone malformations since Si is ubiquitously present in drinking water and foods [4]. Thus, daily Si requirement, based on the amounts consumed and excreted per day, is feasible to reach [6, 10–12]. Nonetheless, the demand of essential minerals, possibly also Si, is higher under certain conditions. During pregnancy, the maternal requirement of minerals is increased due to the growth of and skeletal mineralisation in the foetus [13]. After delivery, maternal demand for minerals continues to stay high if the child is breast-fed [14]. Calcium (Ca), which is one of the most important and a well-studied bone mineral, is highly regulated during these periods [15]. To ensure the greater demand for Ca during pregnancy and lactation, physiological adaptations occur [14]. The primary physiological change during pregnancy is improved efficacy in intestinal Ca absorption [15]. In spite of this, high levels of Ca are detected in the maternal urine due to increased kidneys excretion [14]. Interestingly, and in accordance with Ca, pregnant women also excrete higher levels of Si in urine compared to non-pregnant women [16]. Post-partum, the enhanced intestinal absorption and renal excretion of Ca returns to pre-pregnancy levels and some studies show that Ca excretion decreases further in lactating women [13]. It is not known whether Si excretion changes similarly post-partum. No study has been carried out on Si excretion in post-partum women.

It is difficult to study to what extent the skeleton is used as a mineral source by pregnant women, since ionising radiation, used to measure bone mineral content, should be avoided during pregnancy [13]. However, some prospective studies have managed to measure BMD pre-pregnancy and post-partum [17]. The skeleton appears to contribute to the increased Ca demand during pregnancy [17]. In line with this, increased bone resorption markers in urine and in serum during pregnancy indicates higher bone turnover [15]. Furthermore, there are clear evidence that lactation decreases bone mineral content, particularly at sites with higher trabecular bone content [15]. If Si is an important bone mineral, we hypothesise that the BMD and urinary Ca changes during pregnancy and lactation may also be reflected by changes in urinary Si excretion. Accordingly, the aim of the present study was to investigate, from late pregnancy until 18 months post-partum, if urinary excretion of Si was related to length of lactation and to changes in the bone mineral content.

This study utilised urine samples and BMD measures from a previous study, conducted 2008–2010, investigating changes in BMD during lactation [18]. We also measured urinary Ca concentration in the samples in order to compare with previously published Ca levels, with Si excretion levels, and with BMD changes.

## Subjects and Methods

### Subjects

Pregnant women in South Western Sweden were recruited to participate in research studies investigating changes in bone status and body composition in relation to pregnancy and lactation. The studies were conducted at the University of Gothenburg between 2008 and 2011. Eligible women were recruited through adverts placed at maternal healthcare centres and children’s health centres and their corresponding websites. Inclusion criteria were 25–40 years of age and self-reporting as healthy. Exclusion criteria were use of prescribed medications known to affect bone metabolism, recent bone fractures, pregnancy, or miscarriage (after gestation week 12) in the last 1.5 years before the current pregnancy, breastfeeding during the past year before the current pregnancy, multiple pregnancies, and a current diagnosis of gestational diabetes or preeclampsia.

### Study Design

The participants visited the Department of Internal Medicine and Clinical Nutrition at the University of Gothenburg, Sweden, at gestation weeks 35–37 and at 0.5, 4, 12, and 18 months post-partum. Age, pre-pregnancy body weight, height, smoking habits, parity, and education level were reported. Breastfeeding women were asked at each post-partum time-points if they were still lactating. If not, they were asked to record when lactation had ceased. Following completion of the study, women were divided into groups according to their length of total lactation: 0–3.9 months, 4–8.9 months, and 9 months or longer. The rational for the categorization is that bone mass loss is known to be more pronounced the longer duration of lactation [19, 20]. Total lactation also included, in addition to full breastfeeding, partial breastfeeding in combination with formula feeding.

### Urinary Collection and Analysis

At each time-point (at third trimester and then at 0.5, 4, 12, and 18 months post-partum), participants were asked to collect ~ 10 mL of their first void urine in the morning. At the first study visit, they were supplied with the equipment and instructions for collecting their urine. Participants collected their urine at home the day after the first study visit. Urine was collected in Si-free plastic tubes and were sent by post to the study staff. Urines were then stored at − 20 °C until shipment to Cambridge University (UK) for analysis.

Defrosted urine samples were warmed in a water bath (set at 38 °C) for several hours to re-solubilise any precipitates formed during storage. After cooling and thorough mixing, a 2-mL aliquot of each sample was diluted with 5 mL 0.7% nitric acid diluent, prepared by adding 20 mL of high-purity HNO_3_ (65% UHP grade; Sigma-Aldrich Chemical Co., Gillingham, UK) to 1980 mL of ultra-high-purity water (18 MΩ/cm). A similarly diluted urine sample (1000 mL) was made for preparation of ICP calibration standards. Blank samples consisted of the 0.7% nitric acid diluent alone.

Total analysis for Si and Ca in urine was carried out by Inductively Coupled Plasma Optical Emission Spectroscopy (ICP-OES) using a Jobin Yvon Horiba Ultima 2C ICP-OES (Instrument SA, Longjumeau, France). The ICP-OES was water-cooled and purged with nitrogen gas. The sample introduction system consisted of an integrated peristaltic pump, Conikal U-series Nebuliser (1 mL/min) and cyclonic spray chamber (Glass Expansion, Australia). An AS500 auto-sampler (Horiba Scientific, UK) was also used. The sample introduction pump speed was set to 12 rpm, nebulizer flow rate to 0.84 L/min, and a plasma gas flow rate to 12 L/min. Analysis was by peak profile with a window size of 0.025 nm with 21 increments per profile and an integration time of 0.5 s per increment. Analytical lines for Si and Ca were 251.611 and 317.933 nm respectively.

Calibration standards for Si and Ca (1–56 ppm) were prepared by spiking aliquots (60 mL) of the diluted urine sample with Si and Ca stock ICP standard solutions (1000 ppm Si and 1000 ppm Ca; Merck Ltd, Poole, UK).

The Ultima 2C was set to analyse all samples, blanks, and calibration standards in triplicate and the average value used in the calculation of Si and Ca levels. Si and Ca were analysed sequentially in each sample. A typical batch run consisted of, in the following sequence, blank samples, calibration standards, and then each volunteer’s diluted urine samples (*n* = 2 to *n* = 5 per volunteer) with calibration standards in the middle and at end of batch. The sample introduction system was thoroughly flushed with 0.7% HNO_3_ between samples, blanks, and standards, and extra thoroughly between each volunteer set of samples. A 10-ppm drift check standard was also run after every 20 samples.

For creatinine (Cr) analysis, the urine samples were thoroughly mixed and left to equilibrate and settle at room temperature. An aliquot of each sample was then diluted 1:20 with UHP water (18 MΩ/cm) in a 96-well plate. Fifty microlitres of the diluted sample was then used for Cr analysis. Creatinine analysis was carried out using the Creatinine Urinary Detection Kit (ThermoFisher Scientific Inc., UK). The colorimetric assay was carried out as per the kit instruction/protocol, and plates were read at 490 nm on a FLUOstar Omega Plate Reader (BMG Labtech, UK).

### Bone Mineral Density Analysis

At all post-partum study visits, bone status was measured by dual-energy x-ray absorptiometry (DXA; Lunar Prodigy, software version 11.400.004; GE Healthcare Inc.) at the Osteoporosis Laboratory, Sahlgrenska University Hospital, Gothenburg, Sweden. Areal bone mineral density (aBMD) was measured at several sites, but in the present study, we chose to only include measurements from the lumbar spine (L1–L4) and the femoral neck, because these sites showed the largest changes during the study period [18]. The DXA scanner was calibrated using phantoms. The coefficient of variation for the DXA measurements ranged between 0.5 and 3%.

### Statistical Analyses

The original power analyses [18] revealed that a sample size of at least 15 women in each group was required in order to detect significant differences in changes of aBMD in relation to length of lactation.

Normal distribution of the study participant’s characteristics (age, height, weight, length of lactation) and urinary concentrations of Si (U-Si), calcium (U-Ca), Cr (U-Cr), Si normalised to Cr (U-Si/U-Cr), Ca normalised to Cr (U-Ca/U-Cr), and bone density changes were all assessed with the Shapiro–Wilk test.

Mean and standard deviation of the study participant’s characteristics are presented in Table 1. Significant differences between the lactation groups were tested with one-way analysis of variance (ANOVA) followed by Bonferroni’s multiple comparison test. In the lactation group 0 − 3.9 months, no data are presented for the 18 months post-partum time-point because of insufficient number of samples for statistical analyses.

**Table 1:** Descriptive characteristics of the participating women, grouped based on their length of lactation (a − c)

	0 − 3.9 months of lactation (a)		4 − 8.9 months of lactation (b)		≥ 9 months of lactation (c)		All	
	*N* = 10		*N* = 42		*N* = 29		*N* = 81	
	Mean	SD	Mean	SD	Mean	SD	Mean	SD
Age (y)	30.7^c^	3.2	32.5	3.3	34.3^a^	3.3	32.9	3.4
Height (m)	1.67	0.09	1.69	0.06	1.68	0.06	1.68	0.06
Weight (kg)	73.9	10.1	70.3	10.3	69.4	8.0	70.4	9.5
Length of lactation (mo)	2.3^b,c^	1.5	7.5^a,c^	1.1	11.8^a,b^	2.2	8.4	3.4

Age, height, and weight were recorded 0.5 months post-partum. Descriptive characteristics are presented as mean and standard deviation (SD). Significant differences between the lactation groups were tested with one-way analysis of variance (ANOVA) followed by Bonferroni’s multiple comparison test. Statistically significant difference (*p* < 0.05, adjusted) between lactation groups is denoted by their letter in superscript as ^a^0 − 3.9, ^b^4 − 8.9, and ^c^ > 9 months

**Fig. 1:**
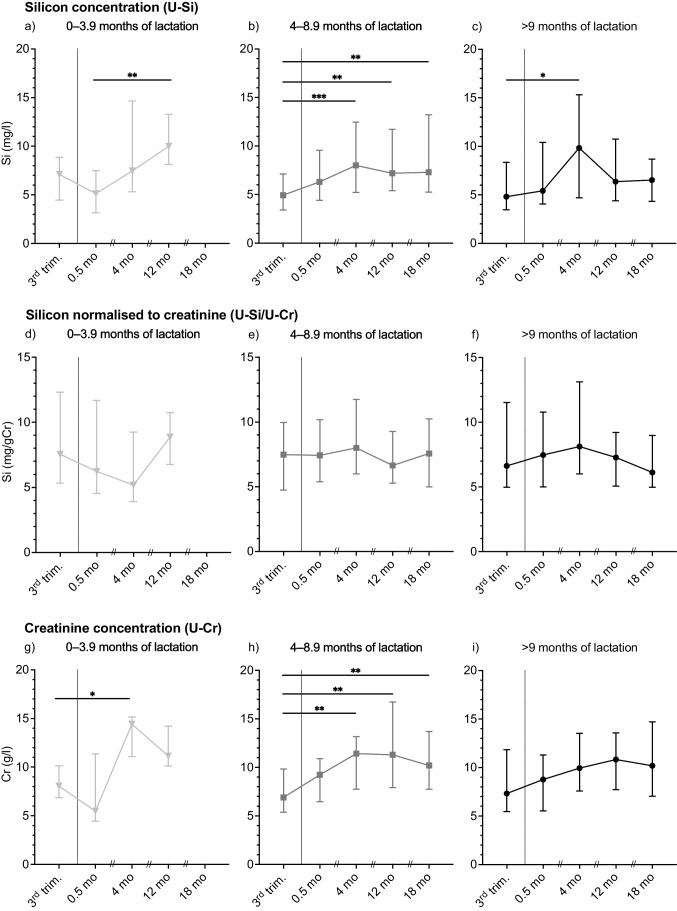
Urinary Si concentration (**a**–**c**; mg/L), urinary Si concentration normalised to creatinine (**d**–**f**; mgSi/gCr), and urinary creatinine concentration (**g**–**i**; g/L) in samples, collected at the third trimester (3rd trim.) and at 0.5, 4, 12, and 18 months (mo) post-partum, from women lactating for 0–3.9 months (**a**, **d**, **g**), 4–8.9 months (**b**, **e**, **h**), and 9 months or longer (**c**, **f**, **i**). The interconnecting line passes through the median value at each time-point, and the error bars indicate the first and third quartiles. Statistically significant differences between time-points within each lactation group were tested with repeated mixed effects analysis followed by Bonferroni’s multiple comparison test; ^*^p < 0.05, ^**^p < 0.01, and.^***^p < 0.001

**Fig. 2:**
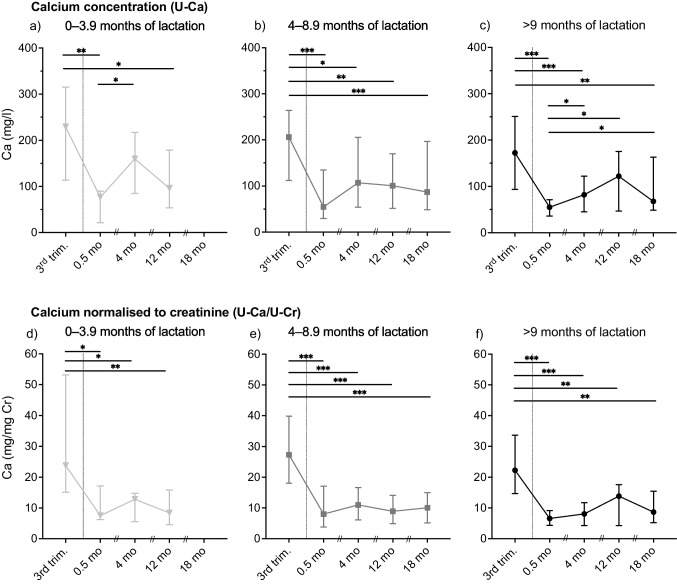
Urinary Ca concentration (**a**–**c**, mg/L) and urinary Ca concentration normalised to creatinine (**d**–**f**; mgCa/mgCr) in samples, collected at the third trimester (3rd trim.) and at 0.5, 4, 12, and 18 months (mo) post-partum, from women lactating for 0–3.9 months (**a**, **d**), 4–8.9 months (**b**, **e**), and 9 months or longer (**c**, **f**). The interconnecting line passes through the median value at each time-point, and the error bars represents the first and third quartiles. Statistically significant differences between time-points within each lactation group were tested with Repeated Mixed Effects analysis followed by Bonferroni’s multiple comparison test; ^*^p < 0.05, ^**^p < 0.01, and.^***^p < 0.001

Since the data for all urinary concentrations showed positively screwed distributions, both the mean and median values, together with the first and third quartiles are presented for U-Si in Table 2, and median with the first and third quartiles in Fig. 1 (U-Si, U-Si/U-Cr, U-Cr) and Fig. 2 (U-Ca, U-Ca/U-Cr). Before statistical analyses, log-transformation of the data was performed to obtain a normal distribution. Statistically significant differences between time-points within each lactation group were then tested with repeated mixed effects analysis followed by Bonferroni’s multiple comparison test. Between groups differences at each time-point were tested with one-way ANOVA. Percentage changes in U-Si, U-Si/U-Cr, U-Ca, U-Ca/U-Cr, and aBMD were calculated as the difference in the log-normal data between each time-point and 0.5 months post-partum followed by multiplication by 100 [21]. Association between the percentage change in U-Si, U-Si/U-Cr, U-Ca, and U-Ca/U-Cr at each time-point and aBMD was tested with Pearson’s or Spearman’s rank correlation coefficient on normally and not normally distributed data, respectively.

**Table 2:** Urinary silicon concentrations (mg/L) in spot-urine samples collected at five different time-points (a − e) from women grouped based on their length of lactation

Time-points	0 − 3.9 months of lactation				4 − 8.9 months of lactation				≥ 9 months of lactation			
	Mean	Median	Q1–Q3	*N*	Mean	Median	Q1-Q3	*N*	Mean	Median	Q1-Q3	*N*
3rd trim. (a)	6.8	7.1	[4.5 − 8.9]	9	5.7^c,d,e^	4.9^c,d,e^	[3.4 − 7.1]	42	7.0^c^	4.8^c^	[3.4 − 8.4]	29
0.5 mo pp (b)	5.4^d^	5.1^d^	[3.2 − 7.5]	9	7.3	6.3	[4.4 − 9.6]	40	8.8	5.4	[4.0 − 10.4]	28
4 mo pp (c)	9.5	7.5	[5.3 − 14.7]	9	9.2^a^	8.0^a^	[5.2 − 12.5]	42	11.5^a^	9.8^a^	[4.7 − 15.3]	29
12 mo pp (d)	11.3^b^	10.0^b^	[8.1 − 13.3]	10	8.8^a^	7.2^a^	[5.4 − 11.7]	41	8.4	6.4	[4.4 − 10.8]	29
18 mo pp (e)	-	-	-	2	9.6^a^	7.3^a^	[5.3 − 13.2]	33	7.4	6.5	[4.3 − 8.7]	21

Silicon concentrations (mg/L) are presented as mean, median, and the first (Q1) and third quartiles (Q3). Data were log transformed before statistical analyses. Within each lactation groups, significant differences between the third trimester (third trim) and 0.5, 4, 12, and 18 months (mo) post-partum (pp) were tested with repeated mixed effects analysis followed by Bonferroni’s multiple comparison test. Within each lactation group, significant difference (*p* < 0.05) between time-points is denoted by the corresponding letter in superscript as ^a^third trimester, ^b^0.5, ^c^4, ^d^12, and ^e^18 months. Statistically significant differences between lactation groups at each time-point were tested with one-way analysis of variance (ANOVA). There were no significant differences in urinary Si concentration between the lactation groups at any time-points

All data analysis and presentations shown were conducted using Prism 9 (GraphPad Software Inc., San Diego, CA, USA). *P* < 0.05 was considered statistically significant in all tests performed.

## Results

### Subjects’ Characteristics

Descriptive characteristics of the participating women, grouped according to their length of lactation, are presented in Table 1. There were no significant differences in weight, height, and body mass index (data not shown) between the lactation groups. Women lactating for 0–3.9 months were significantly younger (*p* = 0.023) compared with the group of women lactating for 9 months or longer. As expected, the mean length of lactation in the three lactation groups, 2.3, 7.5, and 11.8 months, were significant different (*p* < 0.001) to each other.

### Urinary Si Excretion

Urinary Si concentrations (U-Si) in the three different lactation groups are presented in Table 2 and Fig. 1a–c, from the third trimester to 18 months post-partum. Levels were comparable between the three lactation groups, but patterns over the 12/18 months were different. Women lactating for 0–3.9 months had significantly higher (*p* = 0.003) U-Si concentration at 12 months post-partum compared with 0.5 months post-partum (Fig. 1a). Women lactating for 4–8.9 months had significantly higher U-Si concentration at 4 months (*p* < 0.001), 12 months (*p* = 0.003), and 18 months (*p* = 0.006) post-partum, compared with the third trimester (Fig. 1b). For women lactating for 9 months and longer, the U-Si concentration was only significantly higher (*p* = 0.036) at 4 months compared with the third trimester (Fig. 1c). There were no significant differences in U-Si concentrations between the lactation groups at any of the time-points.

The Cr corrected urinary Si excretions (U-Si/U-Cr) are presented in Fig. 1d–f. There were no significant differences in U-Si/U-Cr between the time-points within any of the lactation groups, nor any significant differences between the lactation groups at any time-points.

Urinary Cr concentrations (U-Cr) alone are shown in Fig. 1g–i and in Supplementary Table 1. Women lactating for 0–3.9 months had a significantly higher (*p* = 0.035) U-Cr concentration 4 months post-partum compared with the third trimester (Fig. 1g). U-Cr concentration in women lactating for 4–8.9 months was significantly higher at 4, 12, and 18 months (all *p* < 0.01) post-partum compared with third trimester (Fig. 1h). There were no significant differences in U-Cr concentrations between the three lactation groups at any time-points.

### Urinary Ca Excretion

Urinary Ca concentrations (U-Ca) are presented in Fig. 2. In all three lactation groups, U-Ca concentrations was significantly higher (*p* range < 0.001 to 0.027) at the third trimester compared to post-partum concentrations in all lactation groups, except at 4 months in women lactating for 0–3.9 months (Fig. 2a) and at 12 months in the group of women lactating for 9 months or longer (Fig. 2c). In women lactating for 9 months or longer, U-Ca concentration was significantly lower at 0.5 months (*p* < 0.05) compared with other post-partum time-points (Fig. 2c). There were no significant differences in the U-Ca concentration between the three lactation groups at any of the time-points.

The Cr-corrected urinary Ca excretions (U-Ca/U-Cr) were in all three lactation groups significantly higher (*p* range < 0.001 to 0.022) at the third trimester compared to all post-partum time-points (Fig. 2d–f). There were no significant differences in U-Ca/U-Cr between the lactation groups at any of the time-points.

### Changes in Urinary Si and Ca Excretion and Associations with Changes in aBMD

**Fig. 3:**
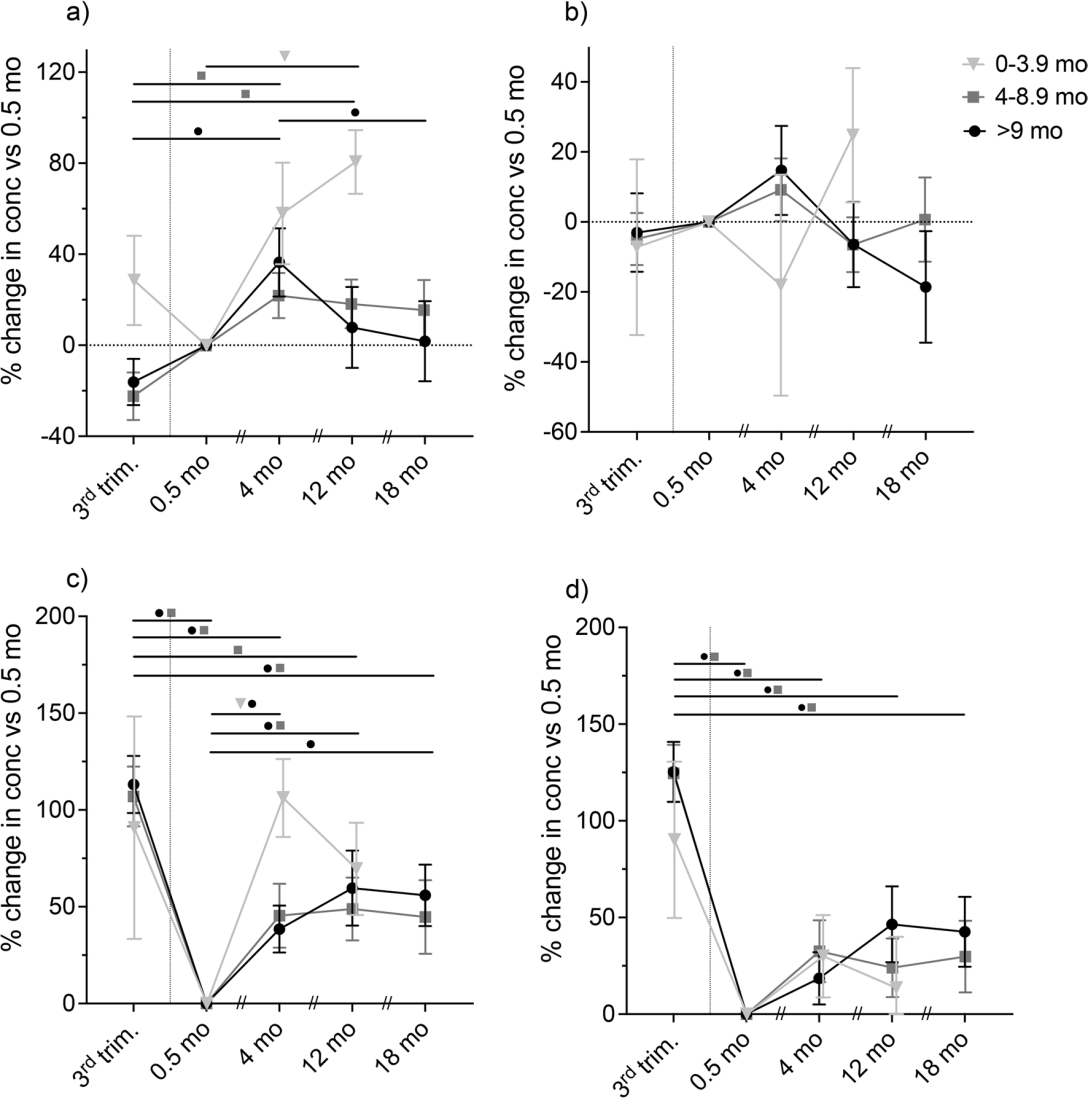
Mean percentage change (± standard error of the mean) in urinary silicon concentration (U-Si; **a**), urinary Si concentration normalised to creatinine (U-Si/U-Cr; **b**), urinary calcium concentration (U-Ca; **c**), and urinary Ca concentration normalised to creatinine (U-Ca/U-Si; **d**) at the third trimester (3rd trim.) and at 4, 12, and 18 months (mo) post-partum compared to 0.5 months in samples from women lactating for 0 − 3.9 months (grey inverted triangle), 4 − 8.9 months (grey square), and 9 months or longer (black circle). Statistically significant differences in change between time-points within each lactation group were tested with repeated mixed effects analysis followed by Bonferroni’s multiple comparison test. Statistically significant difference (*p* < 0.05) is indicated by the corresponding symbol of the lactation group

To account for the heterogeneity within the lactation groups and for further comparison with aBMD, the mean percentage change in Si excretion (U-Si and U-Si/U-Cr), from 0.5 months post-partum to 4, 12, and 18 months post-partum, was calculated for each lactation group (Fig. 3). The percentage change in U-Si concentration in the group of women lactating for 0–3.9 months increased significantly (*p* = 0.003) from 0.5 to 12 months post-partum (Fig. 3a). In the lactation group 4–8.9 months, the change in U-Si concentration was significantly higher at 4 months (*p* < 0.001), and at 12 months (*p* = 0.011) compared with the third trimester (Fig. 3a). In the group of women lactating for 9 months or longer, the percentage change in U-Si concentration was significantly higher at 4 months (*p* = 0.008) compared with third trimester and significantly lower at 18 months (*p* = 0.044) compared with 4 months post-partum (Fig. 3a). There was no significant difference in percentage change in normalised Si excretion (U-Si/U-Cr) at any time-points in any lactation group (Fig. 3b).

The percentage change in urinary Ca excretion (U-Ca) in the groups of women lactating for 4–8.9 months and 9 months or longer was significantly lower at all time-points post-partum (*p* range < 0.001 to 0.013) compared with the third trimester, except at 12 months for the group lactating 9 months or longer (Fig. 3c). Compared with 0.5 months post-partum, the percentage change in U-Ca was however significantly higher at 4, 12, and 18 months (*p* range < 0.012 to 0.047) for the group of women lactating 9 months or longer, significantly higher at 4 months (*p* = 0.01) for the group lactating 0–3.9 months, and at 12 months (*p* = 0.044) for the group lactating 4–8.9 months (Fig. 3c). The percentage change in normalised urinary Ca excretion (U-Ca/U-Cr) was significantly lower at all time-points (*p* range < 0.001 to 0.008) post-partum compared with at the third trimester in the groups of women lactating for 4–8.9 months and 9 months or longer (Fig. 3d).

**Fig. 4:**
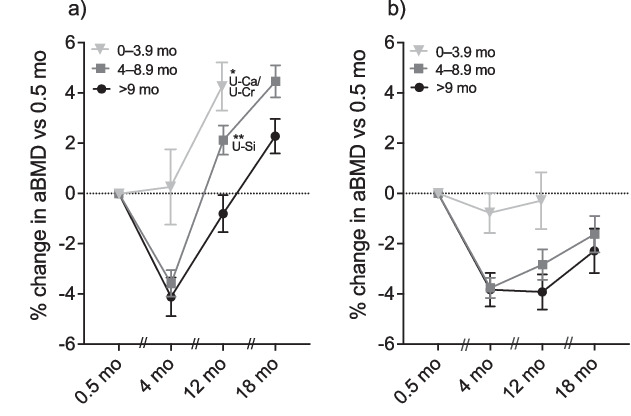
Mean percentage change (± standard error of the mean) in areal bone mineral density (aBMD) at the lumbar spine (**a**) and femoral neck (**b**), at 4, 12, and 18 months (mo) compared to 0.5 months post-partum in women lactating for 0 − 3.9 months (grey inverted triangle), 4 − 8.9 months (grey square), and 9 months or longer (black circle). At each time-point, the relationship between the change in aBMD and the change in urinary Si concentration (U-Si) and Ca concentration (U-Ca) and in urinary Si and Ca concentration normalised to creatinine (U-Si/U-Cr and U-Ca/U-Cr) were tested with Pearson’s or Spearman’s rank correlation coefficient. Statistically significant correlation is indicated as ^*^p < 0.05, ^**^p < 0.01, ^***^p < 0.001, with the corresponding abbreviation

To investigate associations between percentage change in urinary Si and Ca excretion (crude and normalised), and aBMD at the lumbar spine and femoral neck, correlation analyses were performed at each time-point and are marked in Fig. 4 that presents the change in aBMD of the two bone sites. A significant positive association (*r* = 0.48, *p* = 0.002) was found between changes in U-Si concentration and aBMD at lumbar spine between 0.5 and 12 months post-partum in women lactating for 4–8.9 months (Fig. 4a). Furthermore, a significant positive association between changes in U-Ca/U-Cr and aBMD at lumbar spine was found between 0.5 and 12 months (r = 0.47, *p* = 0.027) in women lactating for 0–3.9 months (Fig. 4a).

There were no other significant associations between the percentage change in U-Si, U-Si/U-Cr, U-Ca, U-Ca/U-Cr, and aBMD at the lumbar spine or femoral neck at any other time-points.

## Discussion

Silicon is suggested to be an important nutrient for bone and connective tissue health (1), and dietary Si intake has been shown to be associated with higher BMD (2). Since Si is found to be associated to bone [5, 22], changes in bone metabolism and BMD may thus influence blood Si level and U-Si excretion. Although a previously study reported that blood Si level is low in pregnant women [23], this has not been confirmed [24]. However, in our previous study, we reported higher U-Si excretion in pregnant women compared to non-pregnant women and men [16]. Urinary Si excretion in post-partum and lactating women has not previously been investigated. This longitudinal study investigated, for the first time, changes in U-Si excretion during lactation and whether the changes are associated with the negative changes in aBMD. Changes in U-Ca excretion and the association with aBMD were also investigated.

Lactation has a negative impact on aBMD, and this has previously been confirmed in the participants of the current study [18]. The previous study showed a significant decrease in aBMD at several skeletal sites in women lactating for 4 months or longer [18]. Dividing the participants into groups according to their length of lactation (0–3.9, 4–8.9, and 9 months and longer), as published by Brembeck et al. [18], allowed us to investigate the associations between Si excretion, length of lactation, and aBMD in the present study. Because there were no significant differences in height and weight between the groups, and since the significant difference in age between the group of women lactating for 0–3.9 months and 9 months and longer was only 3.5 years (mean difference), it is unlikely that the anthropological characteristics of the groups are influencing the findings reported here. However, a limitation of the study is that other factors affecting aBMD, such as physical activity and hormonal contraceptives, were not considered.

We measured the U-Ca excretion in order not only to evaluate the accuracy of the analysis by comparing the changes between pregnancy and post-partum with previously published U-Ca changes, but also to compare with the U-Si excretion levels and with aBMD changes. The Ca excretion is reported to increase during pregnancy and subsequently return to pre-pregnancy levels post-partum [13]. In line with this, we found decreased U-Ca excretion post-partum compared with the third trimester, which indicates that the reliability of the analysis of spot urine samples is accurate. The increased excretion levels during pregnancy is suggested to be due to an increased glomerular filtration rate and increased absorption of Ca in the intestine, and possibly the increased bone turnover rate contributes [13]. Despite that there is still an increased demand of Ca in lactating women, reflected by the altered bone mineral status, the U-Ca excretion decreases post-partum because the increased glomerular filtration during pregnancy returns to normal rate [25]. However, there was no association between changes in U-Ca excretion and mineral density in either lumbar spine or femoral neck at any time-point in any of the lactation groups with affected aBMD post-partum (i.e. those lactating > 4 months). Furthermore, the U-Ca excretion did not correspond to the excretion of Si confirmed by statistical correlation analyses (data not shown).

Ten out of the 81 participants ceased lactation at 3.9 months post-partum at the latest. The pattern of U-Si excretion in women lactating for 0–3.9 months was clearly different from the two other groups, but because of the low numbers of participants, it is difficult to make robust conclusions. On the other hand, this group showed almost no change in the aBMD, at any bone site (Brembeck et al.) [18], which is in line with the expected outcome for non-lactating women and women lactating for only a short period. A significant positive correlation between changes in U-Ca excretion and aBMD at the lumbar spine between 0.5 and 12 months was found in this group. However, this was the only association found among all lactation groups at all time-points and can thus be questioned.

Looking at the groups lactating for 4–8.9 months and 9 months or longer, U-Si concentration at 4 months post-partum was significantly higher than at the third trimester (Fig. 1b, c). This holds true even when adjusting for the individual variance in Si excretion and comparing the percentage change in U-Si from third trimester to 4 months post-partum (Fig. 3a). Interestingly, at 4 months, aBMD at the lumbar spine and femoral neck had decreased in these groups (Fig. 4), which may have contributed to the increased Si excretion during the same period [18]. Furthermore, U-Si excretion did not increase further after 4 months in the two groups. Likewise, aBMD did not decrease any further after 4 months. A tendency for an inverse relationship between U-Si and aBMD can thus be implied, but, however, no significant negative correlation between the changes in U-Si excretion and aBMD was found. In contrary, a significant positive correlation was found between U-Si and aBMD at lumbar spine at 12 months when aBMD had recovered from the drop at 4 months (Fig. 4). However, this was the only association found between the changes in U-Si excretion (crude and normalised) and aBMD in all lactation groups at all time-points and thus no thorough interpretation can be made by this finding.

Urinary Si concentrations in the present study were measured in spot urine samples. The gold standard method used to evaluate the excretion of any analyte in urine is to use 24-h urine collections. However, due to its practicality, spot urine sample collection is often preferred, but has its limitation. To reduce the burden of participation for the study population, going through a life-changing period, and thereby increasing the chances of recruiting participants and minimising the number of drop-outs, spot urines were chosen instead of 24-h collections. The disadvantage with spot urines is the concerns that fluid intake will have an influence on analyte excretion/concentration. One way to adjust for the dilution effect is by normalising the analyte concentration with the corresponding U-Cr concentration [26]. Creatinine is a metabolite from the breakdown of the muscles energy reserve phosphorylcreatine and is excreted constantly but in varying concentration and rate over the day [25]. Comparing U-Cr longitudinally, higher concentrations were measured post-partum than at late pregnancy, mainly in women lactating for 4–8.9 months (Fig. 1g–i; supplementary Table 1). Normalising the U-Si concentration to individual U-Cr concentrations, the significant differences seen in U-Si between different time-points within the lactation groups were lost (Fig. 1). Pregnancy affects the renal physiology with increased glomerular filtration rate, which returns to normal levels post-partum [25]. Furthermore, we do not know if muscle metabolism itself is altered during pregnancy and lactation, nor if/how much Cr that is secreted in the breast milk. Thus, the normalisation of U-Si during pregnancy and post-partum may introduce errors in the data. We therefore chose to show both crude (Cr uncorrected) U-Si values, as well as U-Si normalised for U-Cr. Furthermore, in non-pregnant women, the within-individual variance of U-Cr concentration is eightfold higher in spot urine samples compared with 24-h urine collections, which also must be considered in post-partum women [27]. Furthermore, since the crude U-Ca concentrations in the spot urine samples compared well with previously published reports on how U-Ca excretion changes from pregnancy to post-partum [13], this provides validity of using the crude U-Si values.

An essential aspect when discussing mineral metabolism is the dietary intake. A limitation in this study is therefore that we have not evaluated the Si intake. Under normal physiological conditions, a high Si intake would be reflected in higher U-Si excretion [28]. Quantitative information about the participants diet was recorded in a 4-day food dairy prior to each visit, but because the Si content in different foods was not measured and registered in the Swedish National Food Administration database, it was not possible to evaluate Si intake. Whether or not the dietary habits would be reflected in the U-Si concentration is therefore difficult to address. Another concern, regarding the influence of Si intake on the spot urine samples, is how much of the food consumed in the close proximity of time to the collection it reflects. The participants were instructed to collect their first void urines upon waking up in the morning, as their spot urine sample. Depending on when they had their last meal and if they voided their bladder before bedtime, the spot urine sample could either reflect the Si intake from the very last meal before bedtime or the period of fasting after their last meal.

The changes seen in U-Si concentration from late pregnancy to 4 months post-partum in women lactating for 4 months or longer are a novel finding, but because the differences were lost after correcting for Cr excretion, the results are uncertain. On the other hand, since the changes in U-Ca concentrations, measured in the same spot urine samples, are consistent with previous reported findings [13], it supports that the results of the crude U-Si are not irrelevant. To confirm the findings reported here, further studies are needed preferably with 24-h urine collections rather than spot urines. Furthermore, if the results can be verified, the underlying cause of the change in U-Si concentrations needs to be investigated more thoroughly, including taking dietary Si intake into account.

## Conclusion

We conclude that women lactating for 4–8.9 and ≥ 9 months have a significantly higher U-Si concentrations at 4 months post-partum compared with late pregnancy, which was not found in women lactating for a shorter duration. When accounting for U-Cr excretion, no longitudinal differences in normalised U-Si were found. No correlation between changes in Si excretion and aBMD was found, except at lumbar spine from 0.5 to 12 months in women lactating for 4–8.9 months. A limitation of this study is that Si, Ca, and Cr concentrations were analysed in spot urines which are less sensitive to detect changes compared to 24-h urine collections. Taken together, our results suggest that there is a possibility that U-Si excretion increases from pregnancy to post-partum in women lactating for 4 months or longer but are not related to changes in aBMD.

## Supplementary Information

Below is the link to the electronic supplementary material.

Supplementary file1 (DOCX 16 KB)

## Data Availability

The datasets generated during and/or analyzed during the current study are available from the corresponding author on reasonable request.
