# Supplementary material for: Urinary Silicon Excretion in Relation to Lactation and Bone Mineral Density — a Longitudinal Study Post-partum
Source: Biol Trace Elem Res. 2024 Apr 24;203(1):76–87. doi: 10.1007/s12011-024-04175-8 (PMC11700911; doi:10.1007/s12011-024-04175-8)
Supplement: Supplementary file 1 — Supplementary file1 (DOCX 16 KB) [file 12011_2024_4175_MOESM1_ESM.docx]

|  | **0–3.9 months of lactation** | | | | **4–8.9 months of lactation** | | | | **≥ 9 months of lactation** | | | |
| --- | --- | --- | --- | --- | --- | --- | --- | --- | --- | --- | --- | --- |
| Time-points | Mean | Median | Q1-Q3 | N | Mean | Median | Q1-Q3 | N | Mean | Median | Q1-Q3 | N |
| 3^rd^ trim. (a) | 8.2^c^ | 8.0^c^ | [6.9−10.1] | 9 | 7.3^c,d,e^ | 6.9^c,d,e^ | [5.4−9.8] | 42 | 8.3 | 7.3 | [5.5−11.8] | 29 |
| 0.5 mo pp (b) | 7.3 | 5.5 | [4.4−11.4] | 9 | 9.3 | 9.2 | [6.5−10.9] | 40 | 9.4 | 8.8 | [5.5−11.3] | 28 |
| 4 mo pp (c) | 13.4^a^ | 14.4^a^ | [11.1−15.2] | 9 | 10.6^a^ | 11.4^a^ | [7.8−13.2] | 42 | 10.7 | 9.9 | [7.6−13.5] | 29 |
| 12 mo pp (d) | 12.2 | 11.1 | [10.1−14.2] | 10 | 11.7^a^ | 11.3^a^ | [7.9−16.7] | 41 | 10.6 | 10.8 | [7.7−13.6] | 29 |
| 18 mo pp (e) | - | - | - | 2 | 11.0^a^ | 10.2^a^ | [7.8−13.7] | 33 | 10.6 | 10.2 | [7.0−14.7] | 21 |

**Supplementary Table 1.** **Urinary creatinine concentration (g/l)** in spot-urine samples collected at five different time-points (a−e) from women grouped based on their length of lactation.

Creatinine concentrations (g/l) are presented as mean, median and the first (Q1) and third quartiles (Q3).

Data were log transformed before statistical analyses. Within each lactation group, significant differences between the third trimester (3^rd^ trim) and, 0.5, 4, 12, and 18 months (mo) post-partum (pp), were tested with Repeated Mixed Effect analysis followed by Bonferroni’s multiple comparison test. Within each lactation group, significant difference (*p* <0.05) between time-points is denoted by the corresponding letter in superscript as ^a^third trimester, ^b^0.5, ^c^4, ^d^12, and ^e^18 mo. Statistically significant differences between lactation groups at each time-point were tested with One-way analysis of variance (ANOVA). There were no significant differences in urinary creatinine concentration between lactation groups at any time-points.
